# Application of the Support Vector Machine to Predict Subclinical Mastitis in Dairy Cattle

**DOI:** 10.1155/2013/603897

**Published:** 2013-12-25

**Authors:** Nazira Mammadova, İsmail Keskin

**Affiliations:** ^1^Department of Animal Science, Faculty of Agriculture, Siirt University, 56100 Siirt, Turkey; ^2^Department of Animal Science, Faculty of Agriculture, Selçuk University, 42075 Konya, Turkey

## Abstract

This study presented a potentially useful alternative approach to ascertain the presence of subclinical and clinical mastitis in dairy cows using support vector machine (SVM) techniques. The proposed method detected mastitis in a cross-sectional representative sample of Holstein dairy cattle milked using an automatic milking system. The study used such suspected indicators of mastitis as lactation rank, milk yield, electrical conductivity, average milking duration, and control season as input data. The output variable was somatic cell counts obtained from milk samples collected monthly throughout the 15 months of the control period. Cattle were judged to be healthy or infected based on those somatic cell counts. This study undertook a detailed scrutiny of the SVM methodology, constructing and examining a model which showed 89% sensitivity, 92% specificity, and 50% error in mastitis detection.

## 1. Introduction

Mastitis is a primary problem facing dairy herd producers. It influences not only the yield and composition of milk but also the well-being of cows [1]. Early detection of subclinical symptoms has been a goal of research for many years. Mastitis significantly impacts the milk industry in Turkey, as elsewhere in the world [2].

Mastitis, a disease of the udder that is typically the result of bacterial infection, leads to substantial economic losses by reducing milk yields [3–5]. Most mastitis events are subclinical and thus unobserved and untreated, at a level of prevalence which amounts to the submerged part of an iceberg as compared to clinical events. According to Tekeli [6], in herds without an udder health control program, 50% of cows are infected by subclinical mastitis on average, with 2 udder quarters positive for subclinical mastitis. Of mastitis-related economic losses, it is estimated that 20–30% are caused by clinical mastitis, with the remaining losses caused by subclinical mastitis. In 90–95% of cases, the udder and milk appear normal, despite increased somatic cell count (SCC) and decreased milk quality and yield. This long-lasting disease also slows calf development [6].

Various diagnostic tools of mastitis have been proposed, some of which are currently used in the industry [7]. Previously, SCC has been the most widely used measure due to its high correlation with mastitis [7–10]. Nevertheless, doubts exist as to its usefulness as an indicator of intramammary infection [11]. SCC is affected by age, type, lactation rank, milk yield, anatomical and physiological characteristics of udder stress, season, nutrition, shelter conditions, milking technique, and mastitis [12–22]. Electrical conductivity (EC), used to detect changes in milk composition associated with mastitis, is another proven measure which is now used with increasing frequency in the dairy industry. As is well-known, mastitis is associated with increased conductivity of udder tissue, as well as changes in milk's ionic composition. It is likewise associated with decreased levels of certain mineral substances and increased levels of Na and Cl, all of which increase electrical conductivity [23–25]. In modern enterprises that use a computerized herd management system, such valuable quantitative traits as milk yield, milk flow rate, and electrical conductivity are automatically recorded during milking. Such programs flag cows that experience excessive deviation in electrical conductivity as having mastitis. However, these alarms have been observed to be wrong most of the time, so that using EC alone to diagnose mastitis is a suspect practice [2]. Some studies [25–28] have determined that EC could be an indicator of subclinical mastitis when conductivity exceeds 5.5 mS/cm.

Early detection of mastitis is of great importance in terms of improving the quality of milk production, eliminating economic losses, and protecting animal welfare. Using a combination of traits could be a better way to ascertain a cow's health status at any given time. One objective of this research was to use both SCC and EC along with other milk traits to cluster milk samples into two mastitis health categories using SVM. This paper presents a potentially useful SVM-based approach to classify dairy cows into healthy and subclinical mastitis groups.

Vapnik's [29] SVM discriminates input data between two classes by generating a hyperplane that optimally separates classes after input data have been transformed mathematically into a high-dimensional space. SVM is based on the principle of structural risk minimization and has proven to be especially suitable for high-dimension and small sample-size problems. It has two typical applications: classification and regression. SVM has been applied in such classification problems as pattern recognition, text recognition, and protein classification and has yielded high quality results. As a novel machine learning method, SVM has succeeded in solving such complex problems as support vector regression and face recognition tasks [30, 31].

Recently, SVM has been used to automate disease classification, to improve disease detection methods [32], and to address classification problems in many biomedical fields [33, 34]. The SVM algorithm [29, 35] is a classification algorithm that provides state-of-the-art performance in a wide variety of application domains, including handwriting recognition, object recognition, speaker identification, face detection, and text categorization [36]. To date, SVM has been broadly applied in the field of computational biology to address pattern recognition problems, including protein remote homology detection, microarray gene expression analysis, recognition of translation start sites, functional classification of promoter regions, prediction of protein-protein interactions, and identification of peptides from mass spectrometry data [37]. Many biological problems involve high-dimensional, noisy data, for which SVMs are known to behave well compared to other statistical or machine learning methods. In contrast to most machine learning methods, kernel methods such as SVM can easily handle such nonvector inputs as variable length sequences or graphs. Such data types are common in biology applications, often requiring the engineering of knowledge-based kernel functions. Much of this review consists of explaining these kernels and relating them to one another.

The model for the present study was established using the input data of lactation rank (LR), milk yield (MY), electrical conductivity (EC), average milking duration (AMD), and control season (CS). SCC was calculated for milk samples taken once a month over a 15-month period and was used as a system-output indicator of whether an animal was healthy or had subclinical mastitis. Enterprises with large herds do not typically use SCC to gain immediate indications of animals at risk of subclinical mastitis but instead use the data available in the current computing environment.

Predictions based on this approach were compared to those produced by binary logistic regression models containing the same set of variables. A final goal was to illustrate the applicability of the SVM approach by creating a demonstration web-based classification tool. The SVM model enabled the selection of sets of variables that would yield the best classification of cows into the desired groups.

## 2. Materials and Methods

To develop and validate the SVM model for classifying dairy cows into healthy and mastitic groups, data were collected from the KARYEM Private Dairy Farm from February 2010 to April 2011 for 170 Holstein Friesian cattle raised in the KARYEM Agricultural Enterprise situated in the Karapınar district (37°47′ North, 33°35′ East, and 994 m altitude) of Konya province.

To maximize profitability, the KARYEM Agricultural Enterprise uses a professional herd management system that immediately records and stores all events and uses this data to predict developments that could create problems, making all necessary measurements and determinations for this purpose and supplying the manager with all the information thus generated. A user can enter information for individual animals, which can also be registered automatically by the system. The program automatically collects such data derived from the automatic milking system as milk yield, average milking duration, and milk electrical conductivity.

Milking at Karyem occurred twice a day, from 03:00 to 06:00 and from 15:00 to 18:00. Milk samples (50 cc) were carefully collected from the automatic milking system with the help of a sampling apparatus once a month from February 2010 until April 2011 with the primary goal of calculating the milk's somatic cell count (SCC). SCC was calculated in the laboratory via microscope from samples spread over slides and colored. To increase reliability, the count was taken twice for each sample, whereupon the two counts were averaged for data entry.

The SVM algorithm is a new and innovative artificial-intelligence-based method of data mining [35, 38]. SVM can directly distinguish two classes using specific formulas for each. It finds the separator plane that best classifies the data points and separates the two classes of points in the best possible way. In other words, the aim is to find the case of maximal distance between the two classes. The keystone of this classification logic is support vectors, chosen from among training samples that are at the endpoints of both classes.

**Figure 1:**
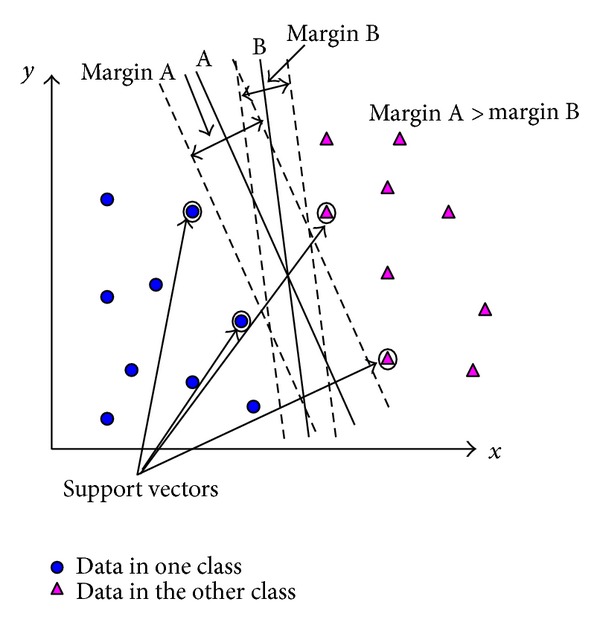
Margin between support vectors in two dimensions.

The goal of SVM modeling is to find the optimal hyperplane that separates clusters of vectors, a set of features, such that data points of one class of the variable are on one side of the plane and those of the other class are on the other. The vectors nearest the hyperplane are the support vectors. In the example of a 2-dimensional case, assume that for classification, the data are from a categorical variable with two classes and there are two prediction variables, *x* and *y*, with continuous values. The data points using the value of *x* on the *x*-axis and *y* on the *y*-axis may be plotted as shown in Figure 1.

**Figure 2:**
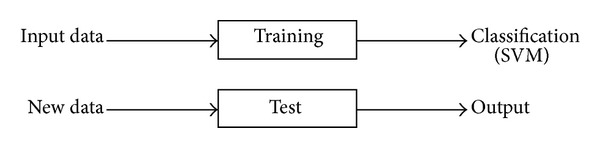
Architecture of classification via SVM.

The figure above is an architecture that applies to all methods of machine learning classifier (Figure 2). During the classification process, part of the available data is reserved for training while the rest is kept for testing, because using training data for classifier accuracy prediction produces good results, that is, higher than those of the actual practices. The ratio of these data directly affects the accuracy rate of the classification process (as well as the error rate). Another factor affecting accuracy rate is data distribution. Mathematically, SVM is based on the structure of the formula regardless of distribution. Moreover, in the SVM technique, the classifier model is created as the SVM is trained after first receiving the training data. Then, the output values that the system will calculate for the test data of the target value are determined. Then, SVM classification performance is evaluated according to the rate of difference between these two values.

Using a training data set, a classification/regression function is set up in SVM. Based on the structural risk minimization principle, SVM focuses on minimizing a bound on the risk function rather than minimizing the error in the training data. Multiple regression models, by contrast, although they have the ability to select specific wavebands, are more useful when there is a linear relationship between the dependent and independent variables, such that SVM is the more appropriate approach for this application.

SVM establishes classification according to the following formula:

(1) $$\begin{array}{c} c=\sum_{i}\alpha_{i}k\left(s_{i},x\right)+b, \end{array}$$

where *s*_*i*_ is a support vector, *α*_*i*_ is the weight, *b* is the bias, and *k* is a kernel function. In the case of a linear kernel, *k* is the dot product. If *c* ≥ 0, then *x* is classified as a member of group 1; otherwise it is classified as a member of group 2.

The SVM regression model for this study was developed using the LIBSVM, a freely available SVM software library [39].

For the binary logistic regression model, the prediction value for each member of the test data set was estimated using the logistic regression function generated during the training step.

The effectiveness of this approach results in high classification accuracy and very good generalization capability. SVM performs effectual classification by mapping input vectors into a higher-dimensional space and constructing a hyperplane that optimally separates the data in the higher-dimensional space. In training, SVM implements a simple linear mapping or linear classifier together with a prior fixed nonlinear mapping in order to make the data separable. This implementation protects training from the problem of local minima and focuses on function optimization with respect to its generalization ability.

The plot shows what classes each (*x*, *y*) point belongs to. In this example, data points of one class are in the lower left corner of the plot and data points of the other classes are in the upper right corner. The SVM analysis has found a one-dimensional hyperplane, that is, a line, to separate the data points based on their target classes. Obviously, an infinite number of such lines are possible. The task of SVM is to determine which line is optimal. For this optimization two parallel lines are constructed, one on each side of the separating line, and the two lines are pushed up against the two sets of data points belonging to the two classes. The distance between the separating line and the closest vectors to the line are labeled. The two lines are used to calculate the margin, defined as the distance between the two. In Figure 1, line A is superior to line B. SVM classifiers are also known as maximum margin classifiers.

Mathematically, suppose that the training data pairs {(*x*_*i*_, *c*_*i*_) | *i* = 1,2,…, *N*} with *x*_*i*_ ∈ *R* ^*n*^ and *c*_*i*_ ∈ {1, −1} indicate what class the data point *x*_*i*_ belongs to. In a simple case, a hyperplane can be defined as *F*(*x*) = *w* ^*T*^ *x* + *w*_0_, where *w* is the adaptable weight vector, *w*_0_ is the bias term, and *T* is the vector transpose operator. In training, the data points *x*_*i*_ are projected into the higher-dimensional space, and the classification is conducted through sign [*f*(*x*)] where *f*(*x*) is the estimate of *F*(*x*). The SVM algorithm searches for a hyperplane *f*(*x*) that maximizes the margin between the two sets of data points in class 1 and −1.

Further, the margin maximum problem can be solved for any high-dimensional space by introducing a kernel function [38, 40]. A nonlinear kernel function allows the low-dimensional input space to be nonlinearly transformed into a high-dimensional feature space such that the probability that the feature space is linearly separable becomes higher. Theoretically, the kernel function can implicitly map the input space into an arbitrary high-dimensional feature space that can be linearly separable even if the input space may not be. Some commonly used kernel functions are the polynomial, the Gaussian, the Sigmoid, and the RBF.

In general, SVM training solves the following optimization problem:

(2) min⁡w,w0,ξ(12wTw+λ∑i=1Nξi)s.t.ci[wTϕ(xi)+w0]≥1−ξi,

where *λ* is a user-defined positive constant; a penalty parameter of the error term, *ξ*_*i*_ ≥ 0, is the slack variable to measure the degree of misclassification of *x*_*i*_; and *ϕ*(*x*) is the function for mapping data points *x*_*i*_ from the input space to a higher-dimensional feature space.

In (2), data points *x*_*i*_ are mapped into a higher-dimensional space using the function of *ϕ*(*x*). Then a linearly separable hyperplane ((*w* ^*T*^ *ϕ*(*x*_*i*_) + *w*_0_)) is found with the maximal margin in the higher-dimensional space. Further, the mapping function *ϕ*(*x*) is determined with a specified kernel function *K*(*x*_*i*_, *x*_*j*_):

(3) $$\begin{array}{c} K\left(x_{i},x_{j}\right)=\phi \left(x_{i}\right)\phi \left(x_{j}\right). \end{array}$$

If RBF is chosen as the kernel function, then

(4) $$\begin{array}{c} K\left(x_{i},x_{j}\right)=\exp\left(-\gamma {||x_{i}-x_{j}||}^{2}\right)\;\left(\gamma >0\right). \end{array}$$

The RBF kernel is well suited to handling nonlinear classification for SVMs.

This study's model was evaluated for sensitivity, specificity, and error rate. The day of observation was classified as true positive (TP) if the output was exceeded on a day of mastitis. A nondetected day of mastitis was classified as a false negative (FN). If no alerts were generated, a day in a healthy period was considered a true negative case (TN) and a false positive case (FP) if an alert was given [41].

Sensitivity represents the number of correctly detected days of mastitis out of all days of mastitis:

(5) $$\begin{array}{c} \text{Sensitivity}\left(\%\right)=\frac{\text{TP}}{\text{TP}+\text{FN}}\times 100. \end{array}$$

Specificity indicates the percentage of correctly found healthy days out of all days of health:

(6) $$\begin{array}{c} \text{Specificity}\left(\%\right)=\frac{\text{TN}}{\text{TN}+\text{FP}}\times 100. \end{array}$$

The error rate is the percentage of days outside mastitis periods of all the days on which an alarm was produced:

(7) $$\begin{array}{c} \text{Error}\left(\%\right)=\frac{\text{FP}}{\text{FP}+\text{TP}}\times 100, \end{array}$$

where TP, FP, TN, and FN represent the number of true positives, false positives, true negatives, and false negatives, respectively.

## 3. Results and Discussion

Milk yields ranged between 14.29 ± 1.680 and 34.20 ± 2.430, electrical conductivity between 3.99 ± 0.097 and 4.50 ± 0.322, average milking duration between 4.38 ± 0.317 and 9.04 ± 1.430, and somatic cell count between 19659 and 471809.

Predictions of early stage subclinical mastitis in Holstein cows using the support vector machine (SVM) techniques are given below.

The five data sets investigated in the present study were classified using the SVM algorithm. In training, 60%, 70%, 75%, and 90% of the total data were used, respectively, while the remaining percentage was considered test data for prediction. The model was trained as it was entered into the mastitis detection (MD) system, with five sets of input data (LR, MY, EC, AMD, CS) and one set of output data. The prediction values obtained were compared with target values. The percentages of the true values were calculated, and the average of the percentage values was obtained after these operations were carried out for each output. The operations were carried out in such a way that no data remained unused in the training and testing stages (Table 1).

The success rates for application of the 60% training-40% test rate for the 5 data sets were 84%, 85%, 88%, 86%, and 83%, as illustrated in Table 1. The success rates of the application of a 90% training-10% test rate were 86%, 77%, 91%, 69%, and 86%, while the success rates of the 75% training-25% test rate application were 84%, 79%, 91%, 81%, and 83%, and the success rates of the 70% training-30% test rate application were 85%, 83%, 91%, 82%, and 84%. At this stage, averages were found for the combination of the four training tests and a result was derived from these averages.

The MD success percentage of the 3rd test results that were observed to be the best of the training results under SVM was 91%.

Of the 346 data collected for 170 cows in the study, 61 were determined to have subclinical mastitis. A lack of subclinical mastitis cases makes disease diagnosis more difficult for the system.

**Figure 3:**
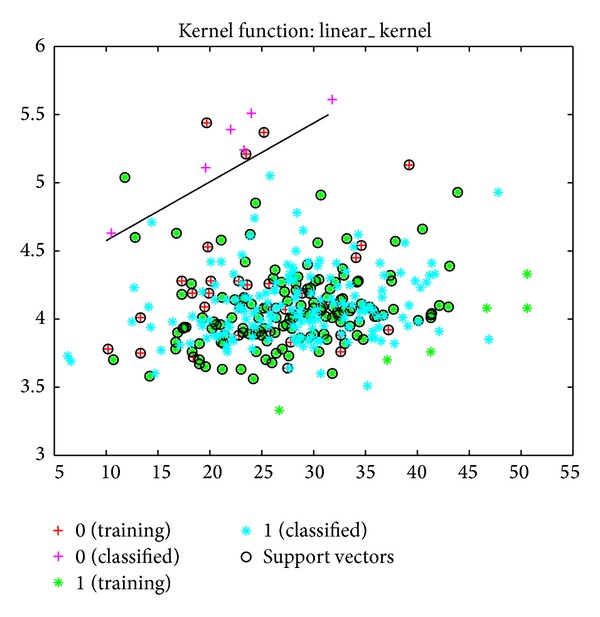
Classification of cows into healthy and subclinical mastitis groups using SVM with kernel function, based on MY and EC data.

**Figure 4:**
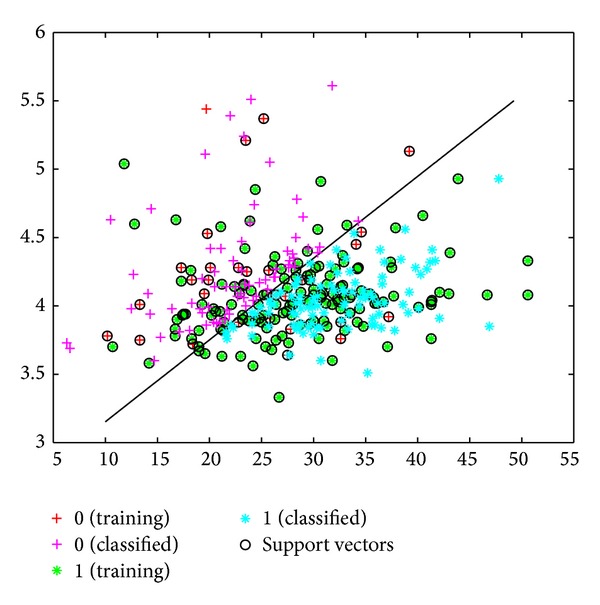
Classification of cows into healthy and subclinical mastitis groups using SVM, based on MY and EC data.

The sensitivity, specificity, and error values for the SVM model were determined to be 89%, 92%, and 50%, respectively. Based on the MY and AMD data, the success rate of classifying healthy cows and cows with subclinical mastitis was 81% (Figure 3). This success rate was 84% when the classification was made using a linear kernel function (Figure 4).

**Figure 5:**
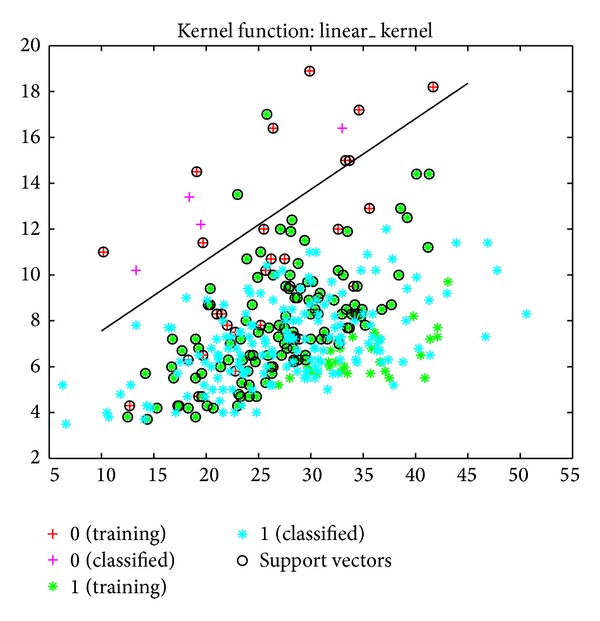
Classification of cows into healthy and subclinical mastitis groups using SVM with kernel function, based on MY and AMD data.

**Figure 6:**
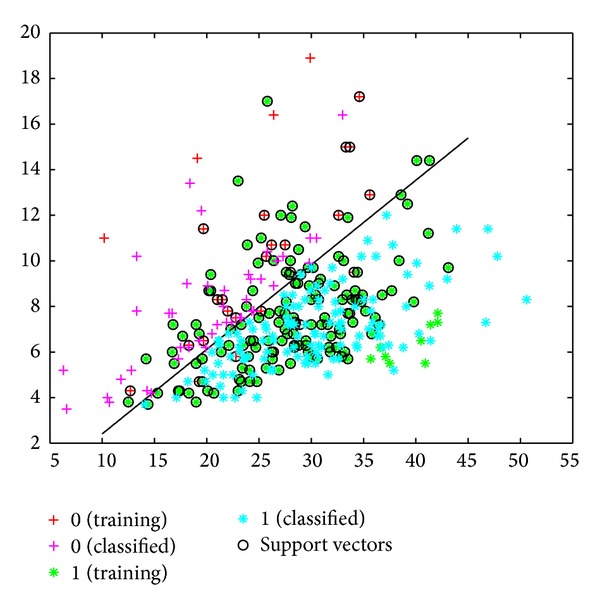
Classification of cows into healthy and subclinical mastitis groups using SVM, based on MY and AMD data.

**Figure 7:**
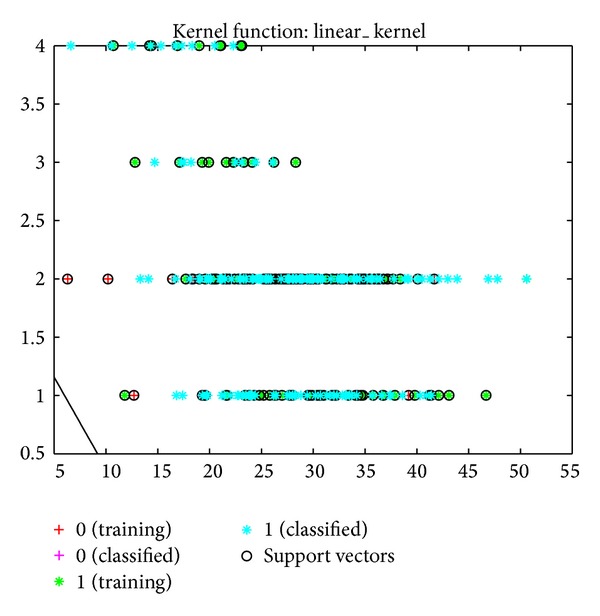
Classification of cows into healthy and subclinical mastitis groups using SVM with kernel function, based on MY and CS data.

**Figure 8:**
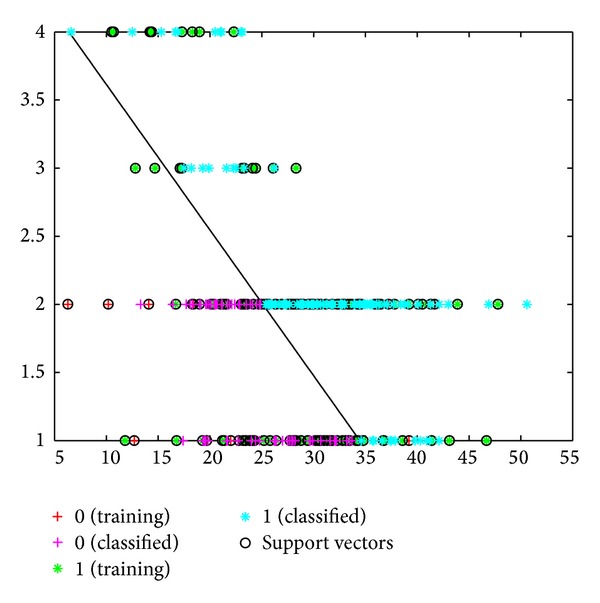
Classification of cows into healthy and subclinical mastitis groups using SVM, based on MY and CS data.

**Figure 9:**
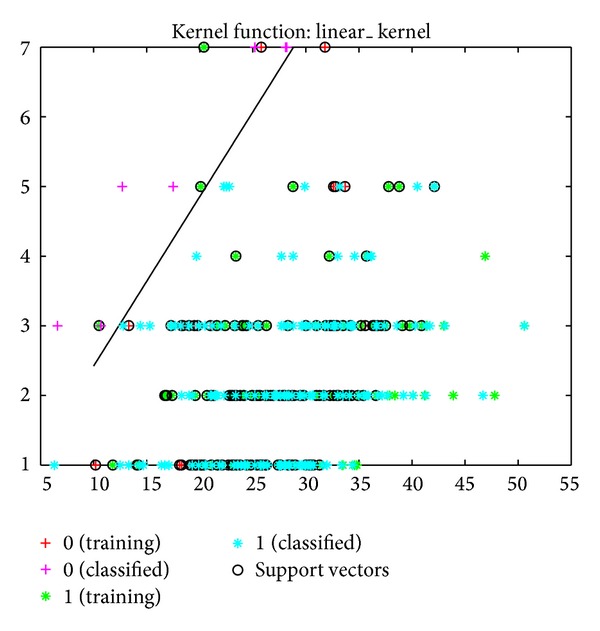
Classification of cows into healthy and subclinical mastitis groups using SVM with kernel function, based on MY and LR data.

**Figure 10:**
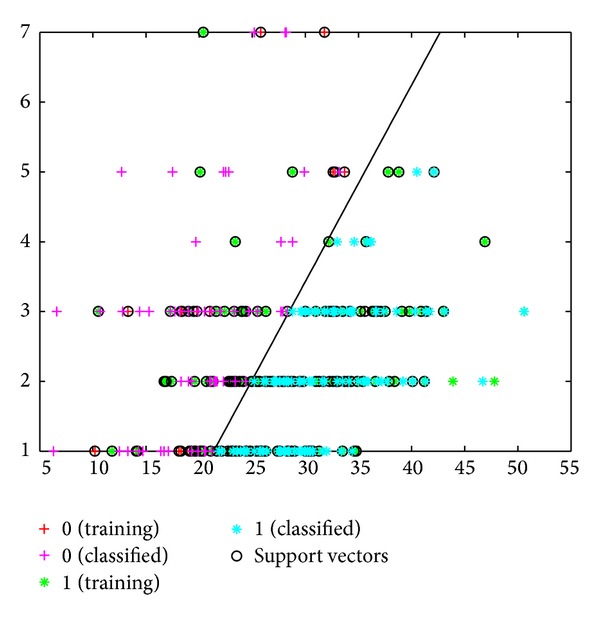
Classification of cows into healthy and subclinical mastitis groups using SVM, based on MY and LR data.

According to the MY and EC data, the success rate of classifying healthy cows and those with subclinical mastitis was 83% (Figure 5). This success rate was 77% when the classification was done using the linear kernel function (Figure 6). The values for the MY and CS versus the MY and LR classification were 83% and 73% as opposed to 82% and 73% (Figures 7, 8, 9, and 10).

The binary logistic regression modeling used the same milk traits and milking variables and used subclinical mastitis detection as the outcome variable. First, the logistic regression analysis was performed for the training data set. Then, the estimated *β* coefficients were applied to the test data set to calculate the probability of each individual being a case. The sensitivity of the model was 75%, its specificity 79%, and its error rate 57%.

## 4. Conclusion

This scheme was an example of the potential use of support vector machine techniques in classifying common diseases in animals. It emerged that the SVM approach can be successfully used to that end and that its classification ability was equivalent to methods now commonly used to detect subclinical mastitis using simple measurements taken by automatic milking systems rather than laboratory tests.

When applied to population health surveys, the SVM technique has the potential to perform better than such traditional statistical methods as logistic regression [42].

The present study's SVM approach, here applied to a data set from Holstein cows, could easily be applied to other animal populations.

Support vector machine modeling thus shows promise in detecting diseases, including subclinical mastitis, even when it uses the simple variables obtained by automated milking systems.

## Figures and Tables

**Table 1:** Results of SVM application.

Total input variable	Training data	Test data	1st test result		2nd test result		3rd test result		4th test result		5th test result		Average	
			Number of true outputs	Success (%)	Number of true outputs	Success (%)	Number of true outputs	Success (%)	Number of true outputs	Success (%)	Number of true outputs	Success (%)			
346	311	35	30	86	27	77	32	91	24	69	30	86	29	82
	260	86	72	84	68	79	78	91	70	81	71	83	72	84
	242	104	88	85	86	83	95	91	85	82	87	84	88	85
	208	138	116	84	117	85	121	88	118	86	114	83	117	85

Average													76	84
